# Production Optimization, Partial Purification, and Biochemical Characterization of an Extracellular Lipase from *Proteus mirabilis* SAB2

**DOI:** 10.1155/ijm/8946340

**Published:** 2026-10-04

**Authors:** Afra M. Baghdadi

**Affiliations:** ^1^ Department of Biological Sciences College of Sciences University of Jeddah Jeddah Saudi Arabia uj.edu.sa

**Keywords:** biochemical characterization, enzyme optimization, extracellular lipase, *Proteus mirabilis*, soil-derived bacteria

## Abstract

Lipases are industrially important biocatalysts with broad applications in biotechnology and environmental processing. In this study, an extracellular lipase‐producing bacterial isolate, SAB2, recovered from soil collected in Rijal Almaa, Saudi Arabia, was identified as *Proteus mirabilis* using phenotypic, biochemical, and 16S rRNA gene sequence evidence. Lipase production was evaluated by a one‐factor‐at‐a‐time (OFAT) approach examining temperature, initial pH, carbon and nitrogen sources, inoculum size, and incubation period. The highest temperature‐series production response was 2.660 U/mL at 20°C; the highest pH‐series response was 2.833 U/mL at pH 3.0, with tar oil as the best‐performing tested carbon source, tryptone as the best‐performing nitrogen source, a 1 mL inoculum, and 9 days of incubation. Because factor interactions were not assessed, these conditions are interpreted as best‐performing OFAT levels rather than a statistically optimized global production condition. The extracellular enzyme was partially purified by 20% ammonium sulfate precipitation followed by dialysis, increasing specific activity from 6.88 ± 0.23 to 39.7 ± 0.69 U/mg, corresponding to 5.77‐fold enrichment and 41.34% recovery. SDS‐PAGE showed a predominant protein band with an apparent molecular mass of approximately 25 kDa. For the partially purified preparation, the highest relative activity was observed at 25°C and pH 5 under the tested assay conditions; activity remained measurable across the tested ranges, with relatively high activity in the presence of Cu^2+^ and Fe^2+^ and lower activity with Hg^2+^ and EDTA. These findings establish *P. mirabilis* SAB2 as a source of an extracellular lipolytic enzyme with activity under relatively mild assay conditions. However, kinetic parameters, preincubation‐based thermal and pH stability, and application‐specific performance remain to be established before claims of cold adaptation, acid stability, or industrial suitability can be made.

## 1. Introduction

Microbial lipases (triacylglycerol acylhydrolases, EC 3.1.1.3) constitute one of the most extensively studied groups of industrial enzymes due to their broad substrate specificity, catalytic efficiency, and ability to function under diverse physicochemical conditions. These enzymes catalyze the hydrolysis of long‐chain triglycerides into free fatty acids and glycerol and, under low‐water conditions, can also mediate esterification and transesterification reactions. Consequently, lipases have found wide‐ranging applications in food processing, detergent formulation, biodiesel production, pharmaceuticals, cosmetics, leather processing, and bioremediation industries. Compared with plant and animal lipases, microbial lipases are particularly attractive because of their extracellular secretion, ease of large‐scale production, genetic manipulability, and cost‐effectiveness [1, 2].

Soil ecosystems, especially those exposed to lipid‐rich substrates, represent a rich reservoir of lipase‐producing microorganisms. Bacteria isolated from soil environments often exhibit adaptive metabolic versatility, enabling them to secrete enzymes with enhanced stability across wide ranges of temperature, pH, and salinity. Among bacterial genera, members of the family Enterobacteriaceae have been frequently reported as efficient lipase producers, owing to their rapid growth rates and ability to utilize diverse carbon sources. Several species belonging to the genus *Proteus* have been documented to produce extracellular lipases with promising biochemical characteristics, making them potential candidates for industrial exploitation [3, 4].

Optimization of culture conditions is a critical step for maximizing microbial lipase production, as enzyme yield and activity are strongly influenced by environmental and nutritional factors such as temperature, pH, carbon and nitrogen sources, inoculum size, and incubation period. Oils and lipid‐based substrates often act as inducers for lipase synthesis, whereas organic nitrogen sources such as peptone, tryptone, and yeast extract have been shown to enhance enzyme production by supporting robust microbial growth. In addition to production optimization, purification and biochemical characterization are essential to evaluate enzyme stability, catalytic performance, and tolerance to metal ions or inhibitors, which collectively determine industrial applicability [5, 6].

Despite the increasing interest in microbial lipases, comparatively limited information is available on extracellular lipase production by environmental *Proteus mirabilis* isolates recovered from Saudi Arabian soils. Importantly, a structurally characterized *P. mirabilis* lipase has previously been assigned to the *Proteus*/psychrophilic subfamily of family I.1 lipases [7]; therefore, the novelty of the present work is not the first demonstration of a *P. mirabilis* lipase. Rather, this study contributes a new soil‐derived isolate, SAB2, and evaluates its extracellular lipase production response to culture variables, followed by partial purification and preliminary biochemical activity profiling. This study is aimed at identifying the isolate, determining the best‐performing levels of individual production variables under submerged fermentation using an OFAT design, partially purifying the extracellular lipolytic preparation, and characterizing its activity under selected physicochemical conditions. Because kinetic and preincubation stability experiments were not performed, the results are interpreted as preliminary biochemical characterization rather than definitive evidence of cold adaptation or acid stability.

## 2. Materials and Methods

### 2.1. Sample Collection and Bacterial Isolation

Soil samples were collected from Rijal Almaa, Kingdom of Saudi Arabia (17°48 ^′^21.9 ^″^N, 42°21 ^′^45.7 ^″^E), using sterile spatulas and transferred into sterile polyethylene bags (Figure S1). Samples were transported to the laboratory under ambient conditions and processed within 24 h. Serial dilutions of soil suspensions were prepared in sterile saline solution (0.85% NaCl), and aliquots were spread onto nutrient agar plates (HiMedia Laboratories, Mumbai, India). After incubation at 37°C for 24–48 h, morphologically distinct colonies were repeatedly subcultured to obtain pure isolates. The isolate exhibiting the highest lipolytic activity was designated SAB2 and selected for further investigation.

### 2.2. Screening for Lipase‐Producing Bacteria

Primary screening for lipase production was carried out using phenol red agar medium (pH 7.4) composed of peptone (5 g L^−1^), yeast extract (3 g L^1^), agar (15 g L^−1^), CaCl_2_ (1 g L^−1^), and distilled water, with pH adjusted using 0.1 M NaOH. After sterilization at 121°C for 15 min, phenol red dye (10 mL L^−1^; 1 mg mL^−1^) and olive oil (10 mL L^−1^) were aseptically added to the cooled medium (approximately 60°C). Plates were inoculated by streaking and incubated at 37°C for 48 h. Lipolytic activity was inferred from localized acidification caused by fatty‐acid release, producing a shift of phenol red from its initial red/pink‐orange appearance toward yellow in the reaction zone [8].

### 2.3. Molecular Identification of the Bacterial Isolate

Phenotypic characterization of isolate SAB2 was performed using colony morphology, Gram reaction, motility, and conventional biochemical tests according to standard bacteriological procedures. The observed phenotypic profile was compared with descriptions in Bergey′s Manual of Systematic Bacteriology.

Genomic DNA of the selected bacterial isolate SAB2 was extracted from overnight cultures grown in nutrient broth using the phenol–chloroform extraction method as described by Sambrook and Russell [9]. The 16S rRNA gene was amplified by polymerase chain reaction (PCR) using the universal bacterial primers 27F (5 ^′^‐AGAGTTTGATCMTGGCTCAG‐3 ^′^) and 1492R (5 ^′^‐TACGGYTACCTTGTTACGACTT‐3 ^′^), originally reported by Weisburg et al. [10]. PCR amplification was performed in a total reaction volume of 50 *μ*L containing 25 *μ*L of 2× PCR Master Mix (Thermo Fisher Scientific, Waltham, Massachusetts, United States), 1 *μ*L of each primer (10 *μ*M), 2 *μ*L of genomic DNA (~50 ng), and nuclease‐free water to volume. Amplification was carried out under the following thermal cycling conditions: initial denaturation at 95°C for 5 min; 35 cycles of denaturation at 95°C for 30 s, annealing at 55°C for 30 s, and extension at 72°C for 90 s; followed by a final extension step at 72°C for 10 min.

PCR products were examined by agarose gel electrophoresis using a 1.2% (w/v) agarose gel stained with ethidium bromide and visualized under UV illumination. The PCR products of the expected size were purified using a QIAquick PCR Purification Kit (Qiagen, Hilden, Germany) according to the manufacturer′s instructions. Purified amplicons were subjected to Sanger sequencing using an Applied Biosystems 3730XL DNA Analyzer (Applied Biosystems, Foster City, California, United States). Sequence reads were compared with sequences in the NCBI GenBank database using BLASTn. The assembled sequence was subsequently submitted to GenBank.

For phylogenetic analysis, the 16S rRNA gene sequence of isolate SAB2 and closely related reference sequences retrieved from GenBank were aligned using ClustalW implemented in MEGA X software [11]. A Neighbor‐joining phylogenetic tree was constructed using the Kimura two‐parameter model, and branch support was evaluated with 1000 bootstrap replications. Species‐level interpretation was based on the combined molecular and phenotypic evidence rather than on phylogenetic placement alone.

### 2.4. Production of Lipase Under Submerged Fermentation

Initial lipase production was established under basal conditions using a medium containing (g L^−1^): peptone, 5; beef extract, 3; NaCl, 2; olive oil, 60; and Tween 80, 10, with pH adjusted to 8.0 before sterilization. A 10% (v/v) inoculum from an overnight nutrient broth culture was transferred into the basal production medium and incubated at 30°C for 3 days with shaking at 120 rpm. These conditions served only as the starting/basal production conditions and should not be confused with the subsequently evaluated OFAT levels. After incubation, bacterial cells were removed by centrifugation at 8000 rpm for 15 min at 4°C, and the clear supernatant was used as the crude enzyme source [5]. Bacterial growth was monitored by optical density at 550 nm using a UV–Vis spectrophotometer (SPECTRO UV–VIS AUTO UV‐2602, LaboMed Inc., Los Angeles, California, United States). The available laboratory record distinguishes these basal conditions from the subsequent OFAT experiments but does not unambiguously document whether the fermentation batch later used for partial purification was produced under the basal conditions or under a combined set of selected OFAT levels; therefore, no claim is made that the purification batch represented a validated multivariable optimum.

### 2.5. Lipase Activity Assay

Lipase activity was determined by titration of fatty acids released from an olive‐oil emulsion. The reaction mixture contained 1 mL enzyme preparation, 10 mL of 10% (v/v) olive oil emulsified in 10% gum acacia, 2 mL of 0.6% CaCl_2_, and 5 mL of 0.2 M phosphate buffer (pH 7.0). The mixture was incubated at 37°C for 1 h with shaking at 100 rpm. The reaction was stopped by adding 20 mL ethanol: acetone (1:1, v/v), and liberated fatty acids were titrated with 0.1 M NaOH using phenolphthalein, with the first persistent pale‐pink endpoint used for endpoint recognition. One unit (U) was defined as the amount of enzyme corresponding to the release of 1 *μ*mol fatty acid per minute under the stated assay conditions [12]. Activity was calculated from NaOH consumption according to: activity (U/mL) = (*V* × *M* × 1000)/(*t* × *v*), where *V* is the volume of NaOH consumed (mL), *M* is the NaOH molarity (mol/L), *t* is the reaction time (min), and *v* is the enzyme volume (mL).

### 2.6. Optimization of Culture Conditions

The effects of temperature (5°C–80°C), initial pH (3–9), carbon source (olive oil, coconut oil, tar oil, and used cooking oil), nitrogen source (peptone, yeast extract, tryptone, ammonium sulfate, casein, and potassium nitrate), inoculum size (1–6 mL), and incubation period (3–9 days) on lipase production were evaluated using a one‐factor‐at‐a‐time (OFAT) approach. Each variable was examined individually to identify the best‐performing tested level; interactions among variables were not assessed. Accordingly, the resulting conditions are not described as a statistically optimized global production condition. Response‐surface or other multivariable designs are recommended for subsequent process optimization. Tar oil was included as an unconventional hydrophobic substrate. Commercial tar‐oil products are available in Saudi Arabia; for example, Yanbu United Company for Trading and Industry (YUNITCO, Yanbu, Saudi Arabia) lists a tar‐oil product with a density of ≥ 900 kg/m^3^ at 15°C, kinematic viscosity of ≥ 1500 cSt at 50°C, flash point (COC) of ≥ 250°C, sulfur content of ≤ 1.5 wt%, water and sediment of ≤ 0.01%, and CS_2_ solubility of ≤ 99.5 wt% (YUNITCO, https://yunitco.com.sa/tar). The supplier, batch number, production route, and compositional certificate of the exact tar‐oil sample used experimentally were not retained in the laboratory record; accordingly, the commercial specification above documents market availability only and should not be interpreted as chemical characterization of the experimental sample.

### 2.7. Purification of Lipase

Crude enzyme was subjected to partial purification by ammonium sulfate precipitation. Solid ammonium sulfate was gradually added to the crude supernatant to achieve 10%–80% saturation with continuous stirring at 4°C. The optimum precipitation was obtained at 20% saturation. The precipitated proteins were collected by centrifugation at 8000 rpm for 1 h at 4°C and dissolved in 0.2 M phosphate buffer (pH 7.0). Specific activity, purification fold, and recovery yield were calculated using the following equations:

• Specific activity (U/mg) = Total activity (U)/Total protein (mg)
• Purification fold = Specific activity (purified fraction)/Specific activity (crude fraction)
• Yield (%) = [Total activity (purified fraction)/Total activity (crude fraction)] × 100

#### 2.7.1. Dialysis

The dissolved protein fraction was dialyzed overnight against the same phosphate buffer at 4°C to remove residual ammonium sulfate using dialysis tubing (molecular weight cut‐off 12–14 kDa).

#### 2.7.2. Sodium Dodecyl Sulphate–Polyacrylamide Gel Electrophoresis (SDS–PAGE) Analysis

Total protein concentration was determined using the Bradford method with BSA as the standard [13]. The protein pattern and apparent molecular‐mass distribution of the partially purified preparation were examined by SDS–PAGE using the discontinuous buffer system described by Laemmli [14]. Enzyme samples (20–30 *μ*g protein) were mixed with 5× SDS sample buffer containing 62.5 mM Tris–HCl (pH 6.8), 2% (w/v) SDS, 10% (v/v) glycerol, and 0.01% (w/v) bromophenol blue, supplemented with 5% (v/v) *β*‐mercaptoethanol under reducing conditions. The samples were heated at 95°C for 5 min prior to electrophoresis.

Electrophoresis was performed on 12% (w/v) polyacrylamide resolving gels with a 4% stacking gel using a Mini‐PROTEAN Tetra System (Bio‐Rad Laboratories, Hercules, California, United States). Prestained protein molecular weight markers ranging from 10 to 180 kDa (Thermo Fisher Scientific, Waltham, Massachusetts, United States) were run in parallel to estimate the molecular mass of the lipase. Following electrophoresis, gels were stained with Coomassie Brilliant Blue R‐250 for 2 h and subsequently destained using a solution of 40% methanol and 10% acetic acid until a clear background was obtained and protein bands were distinctly visible.

#### 2.7.3. Biochemical Characterization of the Purified Lipase

The biochemical activity profile of the partially purified lipase preparation from *P. mirabilis* SAB2 was examined by testing reaction temperature, pH, substrate concentration, enzyme volume, and selected metal ions/inhibitors. These experiments assessed activity under the stated assay conditions and were not preincubation stability experiments. Relative activity was expressed as a percentage of the maximum activity observed within each experiment. Accordingly, the terms thermal stability and tolerance are not used unless residual activity after a defined preincubation exposure was measured.

To determine the effect of reaction temperature on lipase activity, assays were conducted at 5°C, 20°C, 25°C, 40°C, and 80°C, and activity was expressed relative to the highest value observed. These measurements describe temperature‐dependent activity only; they do not establish thermal stability because the enzyme was not preincubated for defined periods at each temperature before a standardized residual‐activity assay. The effect of assay pH was evaluated at pH 3, 5, 7, and 9 under otherwise comparable assay conditions; these measurements likewise describe a pH‐activity profile rather than pH stability.

The effect of substrate concentration on lipase activity was evaluated at 5%, 10%, 15%, and 20% lipid substrate while maintaining the enzyme amount and other assay conditions constant. Similarly, the influence of enzyme amount was examined using 0.5, 1.0, 1.5, and 2.0 mL of the partially purified preparation.

The effects of metal ions and inhibitors on lipase activity were investigated by preincubating the purified enzyme with selected compounds prior to the activity assay. The enzyme was incubated with Cu^2+^, Fe^2+^, and Hg^2+^ ions, as well as the chelating agent EDTA, at a final concentration of 1 mM for 30 min at room temperature. Following incubation, residual lipase activity was measured under standard assay conditions and expressed as a percentage relative to the control assay performed in the absence of additives.

### 2.8. Statistical Analysis

All experiments were performed in three independent experimental runs (*n* = 3), with each independently prepared culture or assay regarded as the experimental unit. Data are presented as mean ± standard deviation (SD). Differences among treatments within each experiment were evaluated by one‐way analysis of variance (ANOVA), followed by Tukey′s multiple‐comparison test at *p* < 0.05 Different lowercase letters in Figures 1 and 2 denote statistically significant differences among treatments. Technical measurements, where applicable, were averaged within each independent experiment and were not treated as separate biological replicates. Because only three independent replicates were available per treatment, statistical inference was interpreted cautiously.

**Figure 1:**
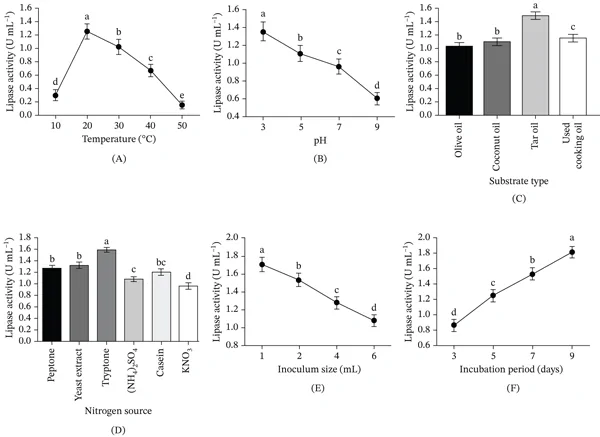
Optimization of cultural conditions for lipase production by *P. mirabilis* SAB2. (A) Effect of temperature, (B) effect of pH, (C) effect of substrate type, (D) effect of nitrogen source, (E) effect of inoculum size, and (F) effect of incubation period. Data are mean ± SD (*n* = 3). Different letters indicate statistically significant differences among treatments (one‐way ANOVA followed by Tukey′s test, *p* < 0.05).

**Figure 2:**
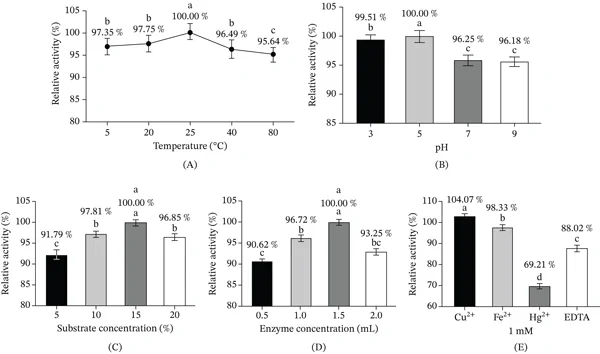
(A) Effect of temperature, (B) pH, (C) substrate concentration, (D) enzyme concentration, and (E) metal ions and EDTA on the activity of partially purified lipase from *P. mirabilis* SAB2. Data are presented as mean ± SD (*n* = 3). Different letters indicate statistically significant differences among treatments (one‐way ANOVA followed by Tukey′s test, *p* < 0.05).

## 3. Results

### 3.1. Isolation and Screening of Lipase‐Producing Bacteria

Soil samples collected from Rijal Almaa, Saudi Arabia, yielded several bacterial isolates following cultivation on nutrient agar. Primary screening on phenol red agar supplemented with olive oil identified isolate SAB2 as a strong lipase producer, as indicated by the formation of a distinct reaction zone accompanied by a localized color change of the phenol red indicator around the inoculation area after 48 h of incubation at 37°C, consistent with lipid hydrolysis and fatty acid release (Figure S2). Based on the intensity and clarity of the reaction zone, isolate SAB2 was selected for subsequent production optimization and biochemical characterization studies.

### 3.2. Identification of the Selected Isolate

#### 3.2.1. Biochemical Profile of bacterial isolate

The selected isolate SAB2 was identified as *P. mirabilis* based on its cultural characteristics and a panel of standard biochemical tests (Table 1). The biochemical profile of isolate SAB2 was consistent with the established characteristics of *P. mirabilis* as described in Bergey′s Manual of Systematic Bacteriology.

**Table 1:** Morphological and biochemical characteristics of *Proteus mirabilis* isolate SAB2.

Characteristic/Test	PX901400.1	Typical reaction for *Proteus mirabilis*
Cell morphology	Rod‐shaped	Rod‐shaped
Gram reaction	Gram‐negative	Gram‐negative
Motility	Positive (swarming)	Positive (swarming motility)
Colony morphology on nutrient agar	Irregular, spreading colonies	Irregular, spreading
Oxidase test	Negative	Negative
Catalase test	Positive	Positive
Indole production	Positive	Negative
Methyl red test	Positive	Positive
Voges–Proskauer test	Negative	Negative
Citrate utilization	Negative	Negative
Urease activity	Strongly positive	Positive
Hydrogen sulfide (H_2_S) production	Positive	Positive
Lactose fermentation	Negative	Negative
Glucose fermentation	Positive	Positive
Gas production from glucose	Negative	Negative
Nitrate reduction	Positive	Positive

The biochemical profile of SAB2 was broadly compatible with the genus *Proteus* and with *P. mirabilis* in several diagnostic characteristics, including Gram‐negative rod morphology, swarming motility, strong urease activity, and negative lactose fermentation. However, the indole‐positive result is atypical for most *P. mirabilis* strains and should not be described as complete phenotypic agreement. The species‐level assignment was therefore based on the combined molecular and phenotypic evidence rather than on the biochemical profile alone.

#### 3.2.2. Molecular Identification

PCR amplification of the SAB2 16S rRNA gene produced the expected amplicon of approximately 1460 bp. Bidirectional Sanger sequencing yielded a 720‐bp partial consensus sequence, which showed ≥ 99% sequence similarity by BLASTn to reference *P. mirabilis* sequences. The sequence is publicly available in NCBI GenBank under accession PX901400.1. In the Neighbor‐joining phylogenetic tree (Figure 3), SAB2 clustered within the *P. mirabilis* lineage and grouped near reference strain OR643825.1. Bootstrap support for the relevant node was 64%, indicating moderate rather than strong support. Accordingly, the phylogenetic tree was considered supportive but not independently definitive for species‐level assignment, and identification of SAB2 as *P. mirabilis* was based on the combined evidence from ≥ 99% BLASTn sequence similarity, phylogenetic placement, and the phenotypic/biochemical profile described above.

**Figure 3:**
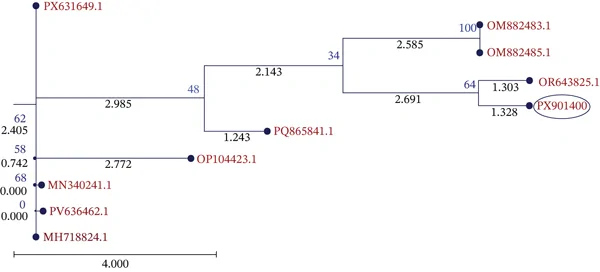
Neighbor‐joining phylogenetic tree of *P. mirabilis* SAB2 based on partial 16S rRNA gene sequences. Bootstrap values (%) from 1000 replicates are shown at the nodes, and the scale bar represents nucleotide substitutions per site. SAB2 (GenBank accession PX901400.1) clusters within the *P. mirabilis* lineage; the 64% bootstrap value indicates moderate support and is interpreted together with BLASTn similarity and phenotypic evidence.

#### 3.2.3. Qualitative Screening of Lipase Activity

Qualitative screening of isolate SAB2 on phenol red agar supplemented with olive oil produced a distinct reaction zone around the inoculation site, consistent with lipid hydrolysis and fatty‐acid release. Local acidification was indicated by a shift of the phenol‐red indicator toward yellow relative to the control (Figure 4), supporting extracellular lipolytic activity and selection of SAB2 for quantitative production experiments.

**Figure 4:**
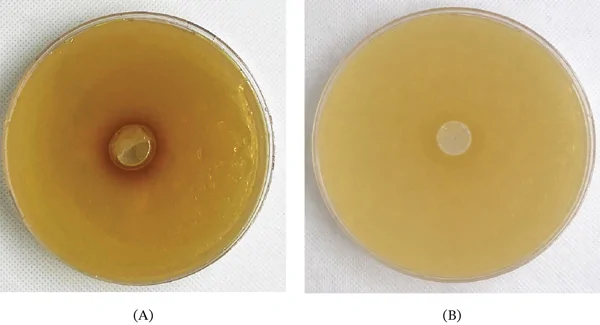
Qualitative screening of extracellular lipolytic activity by *P. mirabilis* SAB2 on phenol red agar supplemented with olive oil. (A) SAB2 showing a localized reaction zone with indicator shift toward yellow, consistent with fatty‐acid release and acidification. (B) Negative control under the same assay conditions.

### 3.3. Effect of Culture Conditions on Lipase Production by *P. mirabilis* SAB2

The individual effects of culture variables on extracellular lipase production by *P. mirabilis* SAB2 are presented in Figure 1. Temperature significantly affected lipase production, with the highest activity observed at 20°C (1.25 U/mL). Activity was lower at 30 and 40°C and declined markedly at 10°C and 80°C (Figure 1A). Initial medium pH also significantly influenced lipase production, with the highest activity recorded at pH 3 (1.35 U/mL), followed by progressively lower activities at pH 5, 7, and 9 (Figure 1B). Among the tested substrate types, tar oil supported the highest lipase production (1.48 U/mL), followed by used cooking oil, coconut oil, and olive oil (Figure 1C). Tryptone was the most favorable nitrogen source, producing the highest activity (1.60 U/mL), whereas KNO_3_ resulted in the lowest response (Figure 1D). Lipase production decreased progressively with increasing inoculum volume, with the highest activity recorded at 1 mL (1.70 U/mL) and the lowest at 6 mL (1.08 U/mL) (Figure 1E). In contrast, lipase activity increased progressively with incubation period, from 0.85 U/mL on Day 3 to a maximum of approximately 1.82 U/mL on Day 9 (Figure 1F). Different letters above the data points or bars indicate statistically significant differences among treatments within each experiment (*p* < 0.05). Collectively, these results identify the best‐performing levels within the individual OFAT experiments; however, they should not be interpreted as a globally optimized combination because interactions among factors were not evaluated.

### 3.4. Partial Purification and Biochemical Characterization of the Lipase from *P. mirabilis* SAB2

#### 3.4.1. Partial Purification of the Lipase

Partial purification of the extracellular lipase produced by *P. mirabilis* SAB2 was achieved using ammonium sulfate precipitation (20% saturation) followed by dialysis (Table 2). The purification process reduced the total protein content from 130.0 ± 2.2 mg in the crude supernatant to 46.0 ± 1.9 mg after ammonium sulfate precipitation and further to 9.32 ± 0.40 mg after dialysis. Although total activity decreased from 895.0 ± 11.0 U in the crude extract to 420.0 ± 9.2 U after precipitation and 370.0 ± 6.8 U after dialysis, the specific activity increased markedly from 6.88 ± 0.23 to 39.7 ± 0.69 U/mg, corresponding to a 5.77‐fold purification with an overall recovery yield of 41.34% in the dialyzed fraction.

**Table 2:** Purification summary of extracellular lipase from *Proteus mirabilis* SAB2.

Step	Total protein (mg)	Total activity (U)	Specific activity (U/mg)	Fold purification	Yield (%)
Crude supernatant	130.0 ± 2.2	895.0 ± 11	6.88 ± 0.23	1.00	100.0
Ammonium sulphate (20%)	46.0 ± 1.9	420.0 ± 9.2	9.13 ± 0.17	1.33	46.93
Dialyzed fraction	9.32 ± 0.4	370.0 ± 6.8	39.7 ± 0.69	5.77	41.34

#### 3.4.2. SDS–PAGE Analysis and Apparent Molecular‐Mass Estimation

SDS–PAGE was used to examine protein enrichment and estimate the apparent molecular mass of the predominant protein band in the partially purified preparation. A prominent band was observed at approximately 25 kDa (Figure S3). Because the preparation was only partially purified and no lipase‐specific zymography, immunodetection, or mass‐spectrometric identification was performed, this band should not be regarded as definitive proof that a single purified 25‐kDa protein is the lipase. The electrophoretic result instead supports enrichment of a predominant protein species; conclusive protein identification will require further purification and/or mass spectrometry.

### 3.5. Effect of Physicochemical Factors on Lipase Activity

The activity profile of the partially purified lipase preparation was evaluated under different assay conditions (Figure 2). The enzyme exhibited its highest relative activity at 25°C, whereas slightly lower activities were retained at 5°C, 20°C, 40°C, and 80°C (Figure 2A). Across the tested pH range, the highest relative activity was observed at pH 5, with comparable activity at pH 3 and modest reductions at pH 7 and 9, indicating a relatively broad pH activity profile (Figure 2B). Substrate concentration produced comparatively small changes in activity, with the highest relative activity observed at 15% substrate (Figure 2C). In the enzyme‐volume experiment, the highest relative activity was recorded at 1.5 mL (Figure 2D). Among the tested additives, activity remained relatively high in the presence of Cu^2+^ and Fe^2+^, whereas Hg^2+^ and EDTA produced greater reductions in relative activity (Figure 2E). These results describe the activity of the partially purified preparation under the tested assay conditions and should not be interpreted as evidence of thermal or pH stability because pre‐incubation stability experiments were not performed.

## 4. Discussion

The present study describes the isolation of a soil‐derived *P. mirabilis* SAB2 strain, evaluation of extracellular lipase production using a OFAT approach, partial purification of the extracellular enzyme, and preliminary characterization of its activity under selected physicochemical conditions. Previous work has established that *P. mirabilis* can produce lipases belonging to the *Proteus*/psychrophilic subfamily of bacterial family I.1 lipases [7]. Therefore, the contribution of the present work lies primarily in the characterization of the environmental isolate SAB2, its extracellular lipase‐production profile, and the biochemical behavior of the partially purified preparation rather than in demonstrating lipase production by the species for the first time.

Phenol red agar provided a rapid qualitative indication of extracellular lipolytic activity. Hydrolysis of lipid substrates releases fatty acids, resulting in local acidification and a corresponding shift of phenol red toward yellow. This assay is therefore useful for preliminary screening but does not provide a quantitative measure of catalytic activity. Quantitative comparisons were subsequently based on the titrimetric lipase assay described in the Materials and Methods section [8, 12].

Culture temperature strongly influenced extracellular lipase production, with the highest response among the tested production conditions observed at 20°C (Figure 1A). This relatively low cultivation temperature may be advantageous for production under mild conditions, but it should not be interpreted as proof of a cold‐adapted catalytic phenotype. Demonstration of cold adaptation requires additional evidence, such as temperature‐dependent catalytic efficiency and residual‐activity measurements after controlled thermal exposure. For comparison, the psychrophilic lipase from *Serratia marcescens* VT1 was characterized using both kinetic and stability experiments and exhibited an optimum reaction temperature of 30°C, stability at 10°C–30°C and experimentally determined K_m_ and V_max_ values [15]. These measurements were not performed for SAB2; accordingly, the present findings support activity under relatively low‐to‐mild temperature conditions but do not establish cold adaptation.

Initial culture pH also influenced extracellular lipase production. The highest production response occurred at pH 3, the most acidic condition tested (Figure 1B), indicating that acidic cultivation favored enzyme production within the OFAT experiment. This production response should be distinguished from the pH dependence of the partially purified enzyme itself. In the biochemical characterization experiment, the highest relative activity was observed at pH 5, whereas substantial activity was retained at pH 3, 7, and 9 (Figure 2B). Thus, the culture pH favoring production was not identical to the pH giving the highest measured catalytic activity after partial purification. Because no defined pH preincubation experiment was performed, these observations describe a pH‐activity profile rather than pH stability.

Carbon source affected extracellular lipase production, and tar oil produced the highest response among the four hydrophobic substrates tested (Figure 1C). This result indicates that SAB2 produced extracellular lipolytic activity under the culture conditions containing this substrate. However, because the experiment measured enzyme production rather than chemical disappearance or degradation of the substrate, the finding should not be interpreted as direct evidence of hydrocarbon biodegradation or bioremediation capability. Such applications would require substrate‐specific chemical analyses and dedicated performance experiments.

Among the tested nitrogen sources, tryptone supported the highest extracellular lipase production (Figure 1D). Organic nitrogen sources can provide peptides, amino acids, vitamins, and other nutrients that support microbial growth and extracellular enzyme biosynthesis. Nevertheless, the OFAT design does not resolve interactions between nitrogen source and temperature, pH, carbon source, inoculum size, or incubation period. Therefore, the apparent superiority of tryptone applies only within the tested experimental conditions and does not by itself establish a statistically optimized medium composition.

Inoculum size also affected lipase production, with the highest activity observed at 1 mL (Figure 1E). The response was not strictly monotonic: activity decreased at 2 mL, increased moderately at 4 mL, and declined again at 6 mL. Incubation time likewise showed a non‐linear pattern, with activity decreasing from Day 3 to Day 5 and then increasing at Days 7 and 9, where the highest response was recorded (Figure 1F). These observations identify the best‐performing levels within individual OFAT experiments. Because factor interactions and a final multivariable validation experiment were not assessed, the selected levels should not be described as a globally optimized production condition. Factorial or response‐surface approaches followed by independent validation would be required for statistical process optimization.

Ammonium sulfate precipitation followed by dialysis increased specific activity from 6.88 ± 0.23 to 39.7 ± 0.69 U/mg, corresponding to 5.77‐fold enrichment with 41.34% recovery. This degree of enrichment is consistent with an early‐stage partial purification. For comparison, a *Pseudomonas aeruginosa* VSJK R‐9 lipase subjected to ammonium sulfate precipitation, dialysis, and subsequent gel filtration reached 11.45‐fold purification with 35.08% yield and an apparent molecular mass of approximately 50 kDa [16], whereas the *S. marcescens* VT1 lipase purified through chromatographic procedures reached 9.42‐fold purification with 16.89% recovery and an apparent mass of 62 kDa [15]. The approximately 25‐kDa band observed for SAB2 should therefore be interpreted as the apparent molecular mass of a predominant protein band in a partially purified preparation, not as conclusive molecular identification of the lipase. Definitive assignment would require additional purification and protein‐level identification, for example by mass spectrometry.

A concise comparison of *P. mirabilis* SAB2 with selected previously characterized bacterial lipases is provided in Table 3. These comparisons should be interpreted cautiously because purification depth, substrates, assay conditions, stability protocols, kinetic characterization, and application testing differ among studies.

**Table 3:** Comparison of *P. mirabilis* SAB2 with selected previously characterized bacterial lipases.

Enzyme/source	Temperature and pH profile	Apparent mass	Purification/yield	Key response and application
*P. mirabilis* SAB2 (present study)	Highest relative activity at 25°C; highest relative activity at pH 5, with substantial activity across pH 3–9. These are activity profiles, not stability measurements.	~25 kDa predominant band; not conclusively identified as the lipase	39.7 U/mg; 5.77‐fold; 41.34% recovery	Relatively high activity with Cu^2+^ and Fe^2+^ and lower activity with Hg^2+^ and EDTA; no application‐specific experiment performed.
*S. marcescens* VT1 [15]	Optimum reaction temperature 30°C; stability reported at 10°C–30°C; pH 7	62 kDa	9.42‐fold; 16.89% recovery	Ca^2+^ and Na^+^ stability reported; EDTA tested; methyl‐ester synthesis demonstrated.
*P. aeruginosa* VSJK R‐9 [16]	Temperature and pH optima not reported in the cited summary used here	~50 kDa	11.45‐fold; 35.08% yield	Restaurant‐wastewater bioremediation evaluated; metal‐ion response not reported in the cited summary used here.
*P. mirabilis* lipase, structural study [7]	Family I.1 Proteus/psychrophilic‐subfamily lipase; optimum temperature and pH were not the focus of the structural study	Crystallographically characterized lipase	Not directly comparable	Ca^2+^ coordination described structurally; prior‐structural evidence for the P. mirabilis lipase family.

The physicochemical experiments describe activity profiles of the partially purified preparation rather than thermal or pH stability. Whereas extracellular production was highest at a cultivation temperature of 20°C, the partially purified preparation showed its highest relative catalytic activity at 25°C in the reaction‐temperature experiment (Figure 2A). Activity remained measurable across the other tested temperatures. Likewise, the highest relative activity in the pH experiment occurred at pH 5, with substantial activity retained across pH 3–9 (Figure 2B). Substrate concentration produced comparatively modest changes in relative activity, with the highest response at 15%, whereas the enzyme‐volume experiment showed its highest relative response at 1.5 mL (Figures 2C,D). These profiles provide preliminary information on assay behavior but do not substitute for formal kinetic characterization or preincubation stability measurements.

The partially purified preparation also retained relatively high activity in the presence of Cu^2+^ and Fe^2+^, whereas lower relative activity was observed with Hg^2+^ and EDTA (Figure 2E). The effect of EDTA may suggest a contribution of metal ions to enzyme structure or catalysis, but this interpretation remains tentative because metal dependence was not investigated mechanistically. Defined chelation and metal‐reconstitution experiments using a more highly purified enzyme would be required before assigning a specific metal requirement or claiming metal tolerance.

Collectively, the data support *P. mirabilis* SAB2 as a source of extracellular lipolytic activity that can be enriched by simple precipitation and dialysis and remains catalytically active under several tested assay conditions. However, no application‐specific experiment was performed. Potential uses in low‐temperature biocatalysis, lipid processing, biodiesel‐related reactions, waste treatment, or bioremediation should therefore be presented as prospective areas for future validation rather than as demonstrated applications of the SAB2 preparation.

Several limitations remain important when interpreting the study. The OFAT production strategy does not quantify interactions among variables or establish a global optimum; preincubation‐based thermal and pH stability experiments were not performed; kinetic parameters such as *K*_*m*_, V_max_, and k_cat_ were not determined; and the approximately 25‐kDa SDS‐PAGE band was not independently confirmed as the lipase protein. In addition, the phylogenetic node supporting SAB2 placement showed only moderate bootstrap support (64%), and the available titrimetric record did not document a matched blank correction. Future work should therefore include multivariable production optimization with independent validation, more complete protein identification and purification, enzyme kinetics, formal stability testing, strengthened taxonomic resolution where needed, and evaluation of a clearly defined application under controlled conditions.

## 5. Conclusions

A soil‐derived bacterial isolate, SAB2, was assigned to *P. mirabilis* on the basis of combined 16S rRNA sequence similarity, phylogenetic placement, and phenotypic evidence, while recognizing the moderate 64% bootstrap support and an atypical indole‐positive phenotype. OFAT experiments showed that extracellular lipase production responded to temperature, pH, carbon source, nitrogen source, inoculum size, and incubation period; the reported levels represent best‐performing conditions within individual experiments rather than a statistically optimized global combination.

The extracellular lipolytic preparation was partially purified by ammonium sulfate precipitation and dialysis, yielding 5.77‐fold enrichment and 41.34% recovery, with a predominant SDS‐PAGE band at an apparent molecular mass of approximately 25 kDa. Activity was measurable across the tested reaction conditions and in the presence of selected metal ions and EDTA. Because the preparation was only partially purified and no lipase‐specific protein identification was performed, the 25‐kDa band cannot yet be assigned definitively as the lipase.

Overall, the study provides preliminary production, partial‐purification, and activity‐profile data for extracellular lipolytic activity from *P. mirabilis* SAB2. The current evidence does not establish cold adaptation, acid stability, kinetic efficiency, or industrial applicability. Future studies should determine enzyme pH‐activity and preincubation pH‐stability profiles, perform thermal‐stability and kinetic analyses, chemically define unconventional substrates, apply multivariable production optimization, increase independent replication where feasible, and evaluate a specific application under controlled conditions.

## Author Contributions

Afra M. Baghdadi conceived and designed the study, performed data interpretation, and wrote and critically revised the manuscript.

## Funding

No funding was received for this manuscript.

## Ethics Statement

This study does not involve human or animal participants; hence, ethical approval was not required.

## Conflicts of Interest

The author declares no conflicts of interest.

## Supporting Information

Additional supporting information can be found online in the Supporting Information section.

## Supporting information


**Supporting Information 1** The following supporting information is available with the online version of this article: Figure S1: Geographic location of the soil sampling site in Rijal Almaa, Kingdom of Saudi Arabia. The sampling point is shown at coordinates 17°48 ^′^21.9 ^″^N, 42°21 ^′^45.7 ^″^E, from which soil was collected for the isolation of the lipase‐producing bacterial isolate SAB2. Figure S2: Primary plate screening of isolate SAB2 on phenol red agar supplemented with olive oil. A localized indicator color change around bacterial growth suggests lipolytic activity. Figure S3: SDS–PAGE analysis of the partially purified lipase from *Proteus mirabilis* SAB2. Lane M represents the prestained protein molecular weight marker (10–250 kDa). The adjacent lane contains the dialyzed enzyme fraction obtained after ammonium sulphate precipitation (20% saturation). A prominent band was observed at approximately ~25 kDa, indicating successful enrichment of the target lipase.

**Supporting Information 2** Table S1: Omnibus one‐way ANOVA and assumption‐test results for OD550 growth measurements obtained during culture‐condition experiments. Table S2: Condition‐level data corresponding to Figure 1: one‐factor‐at‐a‐time culture‐variable profiles for extracellular lipase production by *P. mirabilis* SAB2. Table S3: Condition‐level data corresponding to Figure 2: physicochemical activity profiles of the partially purified lipase preparation from *P. mirabilis* SAB2.

## Data Availability

The data that supports the findings of this study are available in the supporting information of this article.
