# Supplementary material for: Production Optimization, Partial Purification, and Biochemical Characterization of an Extracellular Lipase from Proteus mirabilis SAB2
Source: Int J Microbiol. 2026 Oct 4;2026:8946340. doi: 10.1155/ijm/8946340 (PMC13635783; doi:10.1155/ijm/8946340)
Supplement: Supplementary file 1 [file IJM-2026-8946340-s001.docx]

**Supplementary Tables**

**Table S1A.** Omnibus one-way ANOVA and assumption-test results for OD550 growth measurements obtained during culture-condition experiments.

| Factor | ANOVA F (df1, df2) | Exact P | Shapiro–Wilk W | Shapiro P | Brown–Forsythe F | Brown–Forsythe P | Sensitivity analysis |
| --- | --- | --- | --- | --- | --- | --- | --- |
| Temperature | 1263.92 (4, 10) | 1.796 × 10⁻¹³ | 0.963 | 0.7411 | 0.500 | 0.7368 | — |
| Initial pH | 2713.23 (3, 8) | 2.286 × 10⁻¹² | 0.891 | 0.1218 | 0.407 | 0.7520 | — |
| Carbon source | 588.32 (3, 8) | 1.018 × 10⁻⁹ | 0.882 | 0.0926 | 0.121 | 0.9450 | — |
| Nitrogen source | 2437.18 (5, 12) | 1.328 × 10⁻¹⁷ | 0.924 | 0.1545 | 0.178 | 0.9658 | — |
| Inoculum size | 1137.44 (3, 8) | 7.358 × 10⁻¹¹ | 0.813 | 0.0133 | 0.067 | 0.9761 | Kruskal–Wallis H(3) = 10.495, P = 0.0148 |
| Incubation period | 8250.05 (3, 8) | 2.682 × 10⁻¹⁴ | 0.873 | 0.0716 | 0.143 | 0.9314 | — |

**Note.** One-way ANOVA was applied separately to each culture-variable experiment using the three independent measurements per treatment. Shapiro–Wilk tests were applied to the ANOVA residuals to assess normality, whereas Brown–Forsythe tests were used to assess homogeneity of variances. Residual normality was not rejected for temperature, initial pH, carbon source, nitrogen source, or incubation period (*P* > 0.05), and no evidence of unequal variance was detected by the Brown–Forsythe test for any factor (*P* > 0.05). For inoculum size, the Shapiro–Wilk test indicated departure from residual normality (W = 0.813, *P* = 0.0133); therefore, a Kruskal–Wallis sensitivity analysis was additionally performed and confirmed an overall difference among inoculum-size groups (H(3) = 10.495, *P* = 0.0148).

**Table S2.** Condition-level data corresponding to Figure 3: one-factor-at-a-time culture-variable profiles for extracellular lipase production by *P. mirabilis* SAB2.

| **Panel** | **Culture variable** | **Tested level** | **Mean lipase activity (U/mL)** | **Significance group** |
| --- | --- | --- | --- | --- |
| A | Temperature | 10 °C | 0.30 | d |
| A | Temperature | 20 °C | 1.25 | a |
| A | Temperature | 30 °C | 1.02 | b |
| A | Temperature | 40 °C | 0.67 | c |
| A | Temperature | 80 °C | 0.15 | e |
| B | Initial pH | 3 | 1.35 | a |
| B | Initial pH | 5 | 1.10 | b |
| B | Initial pH | 7 | 0.95 | c |
| B | Initial pH | 9 | 0.60 | d |
| C | Carbon source | Olive oil | 1.03 | b |
| C | Carbon source | Coconut oil | 1.10 | b |
| C | Carbon source | Tar oil | 1.48 | a |
| C | Carbon source | Used cooking oil | 1.15 | c |
| D | Nitrogen source | Peptone | 1.26 | b |
| D | Nitrogen source | Yeast extract | 1.30 | b |
| D | Nitrogen source | Tryptone | 1.60 | a |
| D | Nitrogen source | (NH₄)₂SO₄ | 1.07 | c |
| D | Nitrogen source | Casein | 1.20 | bc |
| D | Nitrogen source | KNO₃ | 0.95 | d |
| E | Inoculum size | 1 mL | 1.70 | a |
| E | Inoculum size | 2 mL | 1.54 | b |
| E | Inoculum size | 4 mL | 1.28 | c |
| E | Inoculum size | 6 mL | 1.09 | d |
| F | Incubation period | 3 days | 0.86 | d |
| F | Incubation period | 5 days | 1.25 | c |
| F | Incubation period | 7 days | 1.53 | b |
| F | Incubation period | 9 days | 1.82 | a |

**Note.** Values are aligned to the final Figure 3 currently used in the manuscript (temperature levels 10, 20, 30, 40, and 80 °C). Means are reported to two decimal places to match the plotted profile. Significance letters reproduce the grouping displayed in Figure 3.

**Table S3.** Condition-level data corresponding to Figure 4: physicochemical activity profiles of the partially purified lipase preparation from *P. mirabilis* SAB2.

| **Panel** | **Assay variable** | **Tested condition** | **Relative activity (%)** | **Significance group** |
| --- | --- | --- | --- | --- |
| A | Reaction temperature | 5 °C | 97.33 | b |
| A | Reaction temperature | 20 °C | 97.75 | b |
| A | Reaction temperature | 25 °C | 100.00 | a |
| A | Reaction temperature | 40 °C | 96.49 | b |
| A | Reaction temperature | 80 °C | 95.64 | c |
| B | Assay pH | 3 | 99.51 | b |
| B | Assay pH | 5 | 100.00 | a |
| B | Assay pH | 7 | 96.25 | c |
| B | Assay pH | 9 | 96.18 | c |
| C | Substrate concentration | 5% | 91.79 | c |
| C | Substrate concentration | 10% | 97.81 | b |
| C | Substrate concentration | 15% | 100.00 | a |
| C | Substrate concentration | 20% | 96.85 | b |
| D | Enzyme concentration | 0.5 mL | 90.62 | c |
| D | Enzyme concentration | 1.0 mL | 96.72 | b |
| D | Enzyme concentration | 1.5 mL | 100.00 | a |
| D | Enzyme concentration | 2.0 mL | 93.25 | bc |
| E | Metal ion / chelating agent (1 mM) | Cu²⁺ | 104.07 | a |
| E | Metal ion / chelating agent (1 mM) | Fe²⁺ | 98.33 | b |
| E | Metal ion / chelating agent (1 mM) | Hg²⁺ | 69.21 | d |
| E | Metal ion / chelating agent (1 mM) | EDTA | 88.02 | c |

**Note.** Relative-activity values and significance letters match the final Figure 4. For panel E, Cu²⁺, Fe²⁺, Hg²⁺, and EDTA are specified at 1 mM, consistent with the corrected Methods and figure labeling. The term “additive” is not used.
