# Supplementary material for: Production Optimization, Partial Purification, and Biochemical Characterization of an Extracellular Lipase from Proteus mirabilis SAB2
Source: Int J Microbiol. 2026 Oct 4;2026:8946340. doi: 10.1155/ijm/8946340 (PMC13635783; doi:10.1155/ijm/8946340)
Supplement: Supplementary file 2 [file IJM-2026-8946340-s002.docx]

**Figure S1.** Geographic location of the soil sampling site in Rijal Almaa, Kingdom of Saudi Arabia. The sampling point is shown at coordinates 17°48′21.9″N, 42°21′45.7″E, from which soil was collected for the isolation of the lipase-producing bacterial isolate SAB2.

**Figure S2**. Primary plate screening of isolate SAB2 on phenol red agar supplemented with olive oil. A localized indicator color change around bacterial growth suggests lipolytic activity.

**Figure S3**. SDS–PAGE analysis of the partially purified lipase from *Proteus mirabilis* SAB2. Lane M represents the prestained protein molecular weight marker (10–250 kDa). The adjacent lane contains the dialyzed enzyme fraction obtained after ammonium sulphate precipitation (20% saturation). A prominent band was observed at approximately ~25 kDa, indicating successful enrichment of the target lipase.
